# Quantitative temporal analysis of pancreatic islet T lymphocyte and macrophage infiltration heralded by serum IgE in congenic BioBreeding (BB) Gimap5^−^/^−^ rats at risk for insulitis and acute onset diabetes

**DOI:** 10.1007/s00011-025-02101-9

**Published:** 2025-10-03

**Authors:** Josefine Jönsson, Linda Faxius, Jeanette Tångrot, Krysten Vance, Stephanie Jerman, Doug Bowman, Marika Bogdani, Peter Ericsson, Rasmus Bennet, Anita Ramelius, Åke Lernmark

**Affiliations:** 1 Department of Clinical Sciences, Skåne University Hospital, Lund University CRC, 214 28 Malmö, Sweden; 2 Department of Molecular Biology, National Bioinformatics Infrastructure Sweden (NBIS), Umeå University, 901 87 Umeå, Sweden; 3 Indica Labs, 8700 Education Pl NW, Bldg. b, Albuquerque, NM 87114 USA; 4 University of Washington, Seattle, WA USA

**Keywords:** Autoimmunity, Beta cell mass, Gimap proteins, IgE, Insulitis, CD3+ lymphocytes, ED1+ macrophages

## Abstract

**Objective and design:**

The objective was to determine the association between serum IgE levels and the infiltration order of T lymphocytes and macrophages in pancreatic islets in relation to the loss of insulin and glucagon cells in presymptomatic congenic BB Gimap5-DP (Diabetes Prone) rats.

**Material:**

Congenic prediabetes BB Gimap5-DP and control Gimap5-DR (Diabetes Resistant) rats were followed every other day from 29 to 32 days of age until peak serum IgE (≤ 55 days of age).

**Methods:**

Serum IgE was measured using ELISA. The HALO™ platform facilitated quantitative image analysis of infiltrating T lymphocytes, macrophages, and target organ insulin and glucagon cells. Whole genome sequencing (WGS) was employed to identify candidate type 1 diabetes genes.

**Results:**

Serum IgE levels increased with age in normoglycemic BB Gimap5-DP rats. Quantification of infiltrating cells per mm^2^ in and around the islets indicated that T lymphocytes are the initial infiltrators, followed by macrophages. Elevated serum IgE levels inversely correlated with beta-cell mass (total mg insulin/mg pancreas). WGS refined the risk segment for islet inflammation to 1.02 Mbp, leaving 10 candidate genes, including *Gimap4* and *Gimap5*.

**Conclusions:**

Elevated IgE levels herald T lymphocyte and macrophage infiltration. Pancreatic islet inflammation was linked to *Gimap4*, *Gimap5,* and other potential candidate genes on rat chromosome 4.

**Supplementary Information:**

The online version contains supplementary material available at 10.1007/s00011-025-02101-9.

## Introduction

The spontaneously diabetic BioBreeding (BB) rat, first described in 1978 [1], is known to develop spontaneous pancreatic islet inflammation that specifically eradicate the beta cells (reviewed in [2–4]). The infiltrates within the pancreatic islets prior to clinical onset were found to represent predominantly dendritic cells [5], macrophages, both CD4+ and CD8+ T lymphocytes, as well as NK cells [6]. The rapid onset of diabetes and almost complete loss of insulin production necessitates stu’dies on the progressive inflammatory process in asymptomatic genetically susceptible rats. Optical projection tomography and morphometry in 40-day-old BBM (M for ‘Malmö’) rats without insulitis revealed a decline in both beta-cell function and mass, along with intra-islet blood flow [7]. Hyaluronic acid deposition in islets was detected prior to insulitis in both humans at risk for type 1 diabetes and presymptomatic BB rats [8]. The islet architecture in humans differs from that in rats, which is similar to mice, complicating direct comparisons (for a review, see [9]). In humans, the inflammatory process occurring in the pancreas prior to the clinical onset has been found to be related to beta-cell autoantibodies directed against either insulin, the 65-kilodalton isoform of glutamic acid decarboxylase (GAD65), Islet Antigen-2 (IA-2), or Zinc transporter 8 (ZnT8) [10, 11]. However, insulitis was reported only in individuals with multiple autoantibodies, not in those with a single autoantibody [10, 12]. Nevertheless, long-term inbreeding and the establishment of a congenic line of rats carrying a frame-shift mutation of the GTPase immune-associated nucleotide-binding protein 5 (Gimap5) gene (BB DR.Gimap5^−^/^−^, hereafter referred to as Gimap5-DP where DP stands for Diabetes Prone) [13–15] have resulted in rats that no longer appear to develop beta-cell autoimmunity against insulin or GAD65 [16, 17] as previously described for outbred BB rats [18, 19]. For clarity, Gimap5-DR (DR for Diabetes Resistant) refers collectively to homozygous wildtype (BB DR.Gimap5^+^/^+^) and heterozygous (BB DR.Gimap5^+^/^−^) rats, both of which are diabetes-resistant. Therefore, the BB rat serves as an attractive animal model since beta-cell killing depends on cytotoxic T lymphocytes without the complicating interference from B cells and autoantibodies. One weakness in BB rat studies has been the lack of a biomarker for insulitis. It has been observed that mast cells and eosinophils may contribute to the initiation of inflammation [20], which was paralleled by increased serum IgE [21]. IgE’s role in type 1 diabetes (T1D) is complex and not fully understood. Profiling of IgE and GAD65 autoantibodies has been utilized to classify T1D into various phenotypes of pathogenesis [22], and newly diagnosed T1D patients, along with their siblings, have exhibited age-related increases in IgE levels [23]. Macrophages and T lymphocytes are the major contributors to islet inflammation [5, 8, 13]; however, their order of infiltration in the pancreas, in relation to age and IgE levels as a possible biomarker of the pathogenesis, remains to be determined.

The aim of the present study was to analyze two congenic lines of BBM Gimap5-DP rats that differed in their reduced frequency of DP genes due to a crossing-over event. We examined the progression of diabetes, performed oral glucose tolerance tests (OGTT), measured serum levels of IgE and other immunoglobulins, and assessed islet autoantibodies in peripheral blood. We tested the hypothesis that IgE levels were related to the temporal infiltration of CD3+ T cells and ED1+ cells (primarily macrophages, but may include some dendritic cells) into the islets. Additionally, we investigated whether the quantities of insulin and glucagon cells correlated with IgE levels along with the temporal pattern of inflammatory cell infiltration.

## Materials and methods

### Animals

**Fig. 1:**
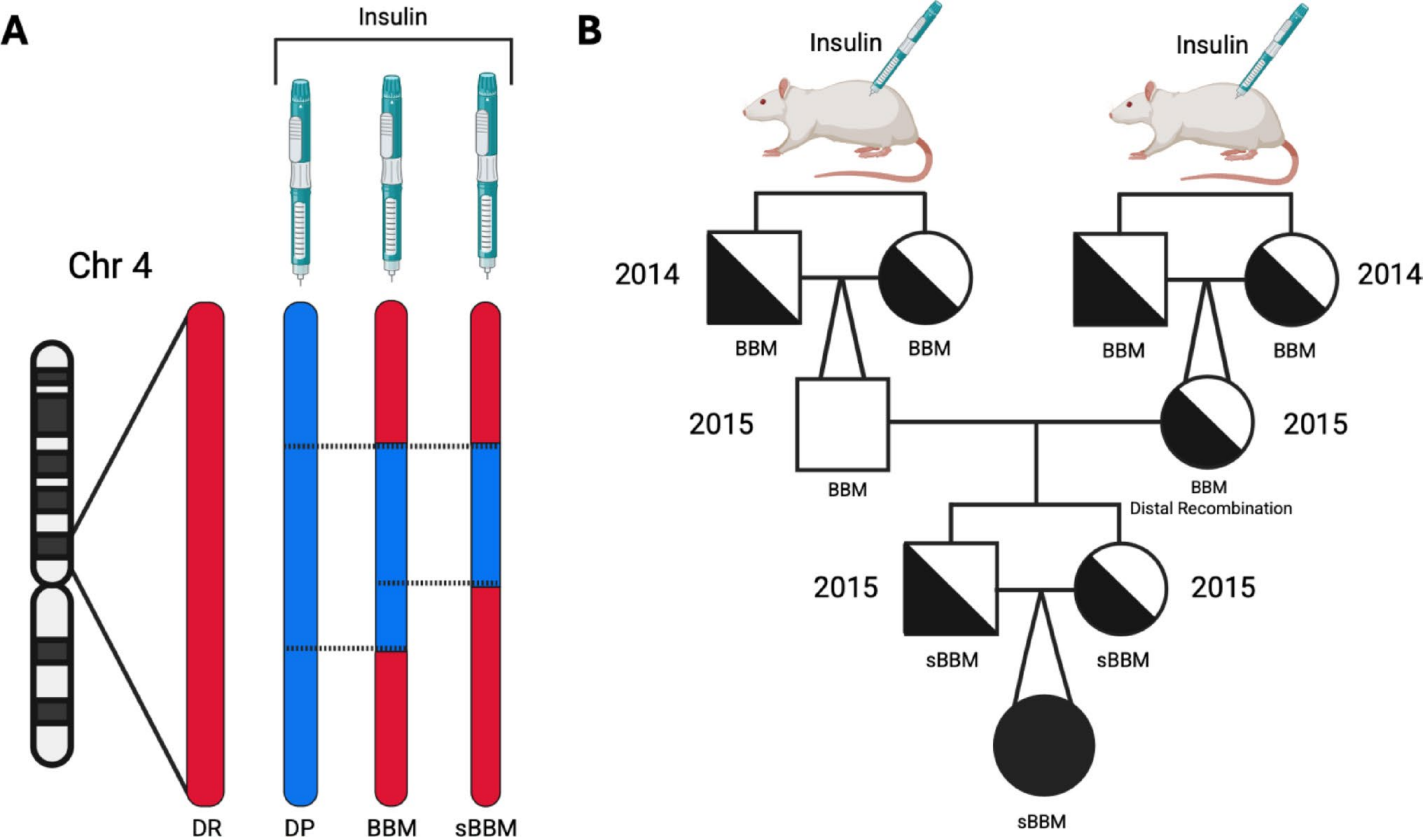
Generation of the sBBM congenic rat strain.** A** Recombination-driven introgression of Diabetes Resistant (DR) DNA (red) into the BBM background to generate the sBBM congenic line. Introgression reduced the Diabetes Prone (DP) rat DNA (blue) contribution on chromosome 4 from 1.44 Mbp to 1.02–1.26 Mbp. **B** Breeding strategy used to establish the sBBM rat strain. A recombination event on the distal end of chromosome 4 (detected in 2013) was rescued through selective breeding, with the desired allele fixed within 2 generations (completed in 2015). The allele was fixed on a uniform genetic background through a strategy of heterozygous sibling breeding, including backcrossing and intercrossing, also completed by 2015. Squares represent males; circles represent females; filled symbols indicate Gimap5^−^/^−^ homozygotes, half-filled symbols indicate Gimap5^+^/^−^ heterozygotes, and open symbols indicate Gimap5^+^/^+^ homozygotes. Two converging lines pointing to a single symbol indicate parents from different litters. Both panels were created using BioRender

Heterozygous congenic BB DR.Gimap5^+^/^−^ rats originated from the colony kept at the University of Washington, Seattle, WA [14, 15], this [17] colony was transferred in 2008 to the Clinical Research Centre (CRC) at Lund University in Malmö, Sweden. The re-derived BB rat line, known as BBM, was confirmed to be free of three Helicobacter strains that were present in the University of Washington colony. For clarity in this study; Gimap5-DP (Diabetes Prone) refers to homozygous mutant rats (Gimap5^−^/^−^), and Gimap5-DR (Diabetes Resistant) refers collectively to homozygous wildtype (Gimap5^+^/^+^) and heterozygous (Gimap5^+^/^−^) rats. Congenic BBM Gimap5-DP rats were obtained through heterozygous sibling breeding. Cross-intercross breeding of congenic BBM Gimap5^+^/^−^ rats was used to reduce the DP residual through recombination events by introgression of DR, aiming to identify T1D risk genes [15]. In addition to the BBM rat, we studied sBBM (with ‘s’ indicating short), which demonstrated a recombination event without affecting the overall phenotype (Fig. 1A). The rats were reared in a Specific Pathogen-Free (SPF) environment at the CRC, Lund University in Malmö, Sweden. They were housed at 21-23ºC with a constant supply of food (brand RM3, SDS (Witham, Essex, England), supplied by Scanbur, Karlslunde, Denmark) and deionized water.

The project was approved by the Animal Ethical Committee in Lund.

### Oral glucose tolerance test

BBM Gimap5^−^/^−^ (Gimap5-DP), Gimap5^+^/^+^ and Gimap5^+^/^−^ (Gimap5-DR) rats, deprived of food for 12 h, were subjected to OGTT (1 g glucose per kg rat) using gavage (PTFE tube,15G, AgnThos AB, Lidingö, Sweden). Blood glucose was measured (Contour® XT, Ascensia Diabetes Care, Basel, Switzerland) at 0 min before gavage and at 5, 10, 15, 30, 60, and 90 min after gavage. Rats with morning blood glucose < 11.1 mmol/L were tested at 40, 45, 50, and 55 days of age.

### Diagnosis of diabetes

Rats were weighed daily, and blood glucose levels were measured from the tail tip every morning (7:30–8:30 AM) starting at 40 days of age. Diabetes was diagnosed when blood glucose reached ≥ 11.1 mmol/L.

### Flow cytometry

Peripheral blood lymphocytes from BBM Gimap5^−^/^−^ (Gimap5-DP), Gimap5^+^/^+^ and Gimap5^+^/^−^ (Gimap5-DR) rats were isolated from one drop of blood collected by puncturing the tail tip with a needle and mixing this drop with 4 mL of phosphate-buffered saline (PBS) containing 10 mmol/L EDTA. The expression of surface markers on peripheral blood lymphocytes was determined using three-color flow cytometry on single-cell suspensions with the following mouse anti-rat monoclonal antibodies (mAb): fluorescein isothiocyanate (FITC)-conjugated CD8a (OX-8); phycoerythrin (PE)-conjugated αβ T-cell Receptor (R73); and phycoerythrin-Cy^TM^5 (PE-Cy^TM^5)-conjugated CD4 (OX-35) (all from BD Biosciences Pharmingen, San Diego, CA, USA).

The R73 antibody recognizes a constant determinant of the rat TCR α/β heterodimer and identifies approximately 97% of peripheral rat T cells [24], thus serving as a reliable pan-T cell marker. Flow cytometric acquisition of the cells was performed on a FACSCalibur (BD Biosciences) and analysed using Cell Quest (BD Biosciences) and FlowJo (Tree Star, OR, USA) software.

### Immunohistochemistry

Rats were killed (Isoflurane Baxter, Baxter, Chicago, IL) and bled terminally from v. cava. The pancreas was weighed, dissected, and fixed for 24 h at room temperature in phosphate-buffered 4% (w/v) paraformaldehyde (Histolab, Gothenburg, Sweden). Serial Sects. (5 µm) were cut at 5 µm to a depth of 500 µm and were placed on SuperFrost® Plus glass, Menzel Gläser (Thermo Scientific, Waltham, MA, USA). The first series of consecutive sections at depths of 5, 30, 55, 215, 240, 265, 425, 450, and 475 µm were stained with Hematoxylin and Eosin to evaluate insulitis. Sections at depths of 15, 40, 65, 225, 250, 275, 435, 460, and 485 µm were stained for CD3, a marker of T lymphocytes (CD3 antibody (clone SP7), diluted 1:500, product no ab 16,669, Abcam, Cambridge, UK) and at 20, 45, 70, 230, 255, 280, 440, 465, and 490 µm were stained for ED1, a macrophage marker (mouse anti-rat CD68, diluted 1:100, MCA341R, Bio-Rad, Hercules, CA, USA). Sections at depths of 10, 35, 60, 220, 245, 270, 430, 455, and 480 µm were double stained with the following primary antibodies: insulin (polyclonal guinea pig anti-insulin; diluted 1:500, product no A0564, Dako, Glostrup, Denmark); glucagon (monoclonal anti-glucagon; diluted 1:2000, product no. G 2654, Sigma-Aldrich, Merck, Saint Louis, MO, USA).

### CD3 and ED1 staining

Sections were deparaffinized, and antigens were unmasked. Next, the sections were incubated for 1 h with primary antibodies diluted in PBS supplemented with 1% (w/v) BSA. The secondary antibody for CD3 was incubated for 30 min using the ImmPRESS® Horse Radish Peroxidase (HRP) Horse Anti-Rabbit IgG Polymer Detection Kit, Peroxidase (MP-7401, Vector Laboratories, Newark, CA, USA). The secondary antibody for ED1 was incubated for 30 min with the ImmPRESS® HRP Horse Anti-Mouse IgG, Rat adsorbed Polymer Detection Kit, Peroxidase (MP-7422, Vector Laboratories). The CD3 and ED1 antibodies were visualized for 6 min using the ImmPACT® DAB Substrate Kit and peroxidase (SK-4105, Vector Laboratories). CD3 staining required a preheated solution of Methyl Green (H-3402, Vector Laboratories) for 10 min and a 1-min rinse in deionized water before dehydration and mounting in Pertex® (Histolab) The ED1 stained sections were counterstained for 3 min in Mayers HTX, followed by dehydration and mounting in Pertex® (Histolab).

### Insulin and glucagon staining

Sections were deparaffinized, and endogenous peroxidase and alkaline phosphatase activity were reduced. After a short rinse in PBS, the sections were incubated for 20 min with normal goat serum (2.5%), followed by 1 h with the insulin antibody diluted in PBS with 2.5% (w/v) BSA. After rinsing in PBS, the slides were incubated for 30 min with the secondary antibody, goat anti-guinea pig IgG (H + L) HRP (1:500) (A18769, Invitrogen™, Thermo Fisher Scientific, Sweden). The sections were rinsed in PBS, and ImmPact DAB (peroxidase substrate kit SK-4105, Vector Laboratories), prepared according to the manufacturer’s instructions, was used for 7 min to visualize the insulin antibody. Following a 5-min rinse in PBS, the sections were further incubated first for 20 min in 2.5% horse serum and then for 1 h with the glucagon antibody, diluted in PBS, rinsed, and followed by a 30-min incubation with the second antibody (ImmPress® AP horse anti-mouse IgG Polymer Detection Kit MP-5402, Vector Laboratories). After rinsing in PBS for 5 min, sections were incubated for 10 min with Vector Blue (SK-5300, Vector Laboratories) to visualize the glucagon antibody. Counterstaining was performed for 10 min in a preheated solution of Methyl Green (H-3402, Vector Laboratories) at 60 °C, rinsed in deionized water for 1 min, dehydrated, and mounted with Vectamount® Permanent Mounting Media (H-5000-60, Vector Laboratories).

### Image analysis

Quantitative image analysis was performed using the HALO™ platform (Indica Labs Inc., Albuquerque, NM, USA), which includes AI-based tissue classification, cell quantification, and spatial analysis modules. Sectioned images were annotated to exclude artifacts (e.g., folds, necrotic tissue, out-of-focus areas). A deep-learning-based tissue classifier was trained to distinguish islets from exocrine pancreatic tissue.

CD3+ and ED1+ cells were quantified using nuclear segmentation and classified as positive or negative based on predefined intensity thresholds. The density and percentage of positive cells were measured within the islets and throughout the entire pancreas. Spatial analysis [25, 26] quantified immune infiltration in 10 µm increments across 100 µm both inside and outside the islet boundary.

The quantification of insulin- and glucagon-positive areas was performed using a brightfield IHC area quantification algorithm. Outputs included stain intensity distribution (0, 1+ , 2+ , and 3+), total positive area, and optical density per stain. The neural network-identified islets enabled separate measurements of stain-positive area in islets versus the surrounding pancreas.

Islet weights were estimated by extrapolating area fractions to the pancreas wet weight in milligram, as described [27], without adjusting for body weight. Similarly, insulin- and glucagon-positive pancreatic tissue (in milligrams) was estimated by multiplying the proportion of positive area on histological sections by the total pancreas weight. Nine histological sections spanning the entire pancreas were analyzed per rat and stain—(i) hematoxylin and eosin, (ii) CD3, (iii) ED1, and (iv) insulin and glucagon—resulting in a total of 36 sections per pancreas. The proportional islet area was calculated for each section, and the mean across sections was used as the representative value for each pancreas. For simplicity, equal tissue density was assumed between islets and exocrine pancreas when converting area measurements into relative weight estimates. This approach allowed conversion of histological area measurements into approximate tissue weights for comparative quantitative analysis.

### Insulitis score

Insulitis was evaluated in hematoxylin- and eosin-stained sections of the pancreas as previously described [13].

### Serum total immunoglobulin isoforms

Samples from three study groups were analyzed: 1) longitudinal sampling involving every other day bleeding of rats followed from 38 days of age until diabetes onset were analyzed for total IgE, IgG, IgA, and IgM; and 2) cross-sectional sampling from rats killed between 20 to 50 days of age and at diabetes onset were analyzed for IgE and Eotaxin; 3) longitudinal sampling every third day from 29 days of age for the analysis IgE until a high IgE level was reached but prior to diabetes. Each immunoglobulin isoform was measured in serum (100 μl in each assay) using isoform-specific ELISA kits ( OKIA00152 IgE, OKIA00150 IgA, OKIA00153 IgG, and OKIA00154 IgM, Aviva Systems Biology, San Diego, CA). Eotaxin levels were measured by ELISA (Legend Max rat CCL11; cat no 444007, BioLegend, San Diego, CA). The analyses were carried out in duplicates according to the manufacturer’s instructions. The coefficient of variation for duplicate samples was 3.8%.

### Islet autoantibodies

Autoantibodies against truncated GAD65 [28] (tGADA), GAD65 (GADA), IA-2 (IA-2A), and ZnT8 (ZnT8A) were determined using a standard radiobinding assay [29] as described in detail elsewhere [30]. In the present assays, Sepharose conjugated with Protein G (Merck, Darmstadt, Germany) was used instead of Protein A-Sepharose to separate the free antigen from the antibody-bound labeled antigen [29].

The intra-assay coefficient of variation for duplicates was 24% for tGADA, 30% for GADA, 18% for IA-2A, and 14% for ZnT8A.

### Genotyping

The rats were genotyped using seven single nucleotide polymorphisms (SNPs) from earmarking biopsies, as detailed elsewhere [13]. These SNPs mark the presence of either BB-DP or BB-DR DNA, allowing for the identification of recombination events in the BBM Gimap5-DP rats to shorten (s) the DP DNA contribution in the novel sBBM congenic line.

### Whole genome sequencing

One Gimap5^+^/^+^ (Gimap5-DR) and one Gimap5^−^/^−^ (Gimap5-DP) rat from the sBBM congenic line were whole genome sequenced at the National Genomics Infrastructure (NGI)/SciLifeLab (Uppsala, Sweden). Two sequencing libraries were prepared from each rat using the TruSeq PCR-free DNA Library Preparation Kit (Illumina Inc., San Diego, CA, USA), and paired-end sequencing was performed using an S4 flow cell on the NovaSeq 6000 system with v1.5 sequencing chemistry (Illumina Inc.).

### Read mapping and per-sample variant calling

General quality control (QC), mapping, and variant calling were performed using the nf-core [31] pipeline Sarek [32] version 2.7 [10.5281/zenodo.3476426] and Nextflow [33] version 20.10.0. Reads were mapped to the Rnor_6.0 reference genome with BWA-MEM [34] version 0.7.17-r1188. Genome Analysis Tool Kit (GATK) [35, 36] version 4.1.7 was used to mark duplicates and perform base quality score recalibration (BQSR) before calling variants with GATK HaplotypeCaller.

Samtools stats [34] version 1.9 and Qualimap bamqc [37] version 2.2.2-dev, were used for preprocessing quality control, while MultiQC version 1.8 provided an overall summary of the run and the QC results.

### Variant annotation, filtering, and comparison

Variant Effect Predictor (VEP) version 99 [38] was used to annotate the variants, including predictions of the effects of each variant. Variants were initially quality filtered using GATK VariantFiltration [35, 36] and the same GATK instance employed by Sarek. Hard filters were applied since no truth set is available for rats. Filters were applied separately for SNPs and insertion-deletions (INDELs). The filtering criteria for SNPs were: QD < 2.0, QUAL < 30.0, SOR > 3.0, FS > 60.0, MQ < 40.0, MQRankSum < −12.5, and ReadPosRankSum < −8.0, while the criteria for INDELs were: QD < 2.0, QUAL < 30.0, FS > 200.0, and ReadPosRankSum < −20.0. After filtering, SNPs and INDELs were merged back into the same VCF file, retaining only the variants that passed all filters.

The region of interest (region 77,280,000–79,360,000 on chromosome 4) was extracted using tabix from samtools [39] version 1.13, and the variants in this region were further filtered based on the annotations using the VEP tool filter vep. Only variants expected to affect protein-coding regions, specifically those in protein-coding transcripts, were retained, with the predicted consequences required to include splice donor variant, splice_acceptor variant, stop gained, frameshift_variant, missense variant, stop lost, or initiator codon variant.

### Statistics

The individual data points in the scatter and line plots represent the levels of the variable of interest (serum IgE levels vs. age, weight of insulin or glucagon-positive pancreas vs. serum IgE levels, and eotaxin vs. age) in a specific sample. Statistical testing was conducted based on the average level of the variable of interest in the sections for each rat on a particular day. In regards to 2) the cross-sectional sampling and 3) longitudinal sampling every third day, serum IgE levels or Eotaxin were modeled using linear regression. In the first analysis, a model without an interaction term was fitted with serum IgE or eotaxin as the dependent variable and genotype and age (in days) as independent variables. This model assessed overall serum IgE or eotaxin differences between genotypes while assuming a common age-related slope across groups. In a second analysis, an interaction term between genotype and age was added to the model. This interaction model allowed us to test whether the rate of increase in IgE with age differed between genotypes. To account for the correlation between multiple samples taken from the same rat’s pancreas (serial sections), mixed effects modeling was employed to ascertain the statistical significance levels when comparing the weights of insulin or glucagon in the pancreas to the serum IgE levels.

The correlation was estimated using the average expression per animal derived from multiple sequential slides. The samples were stratified by rat genotype, and Pearson’s correlation was estimated for all pairwise combinations of variables. Only significant (*p* < 0.05) values are shown in the correlation matrices.

We performed mixed-effects models (R-package *lme4*) to assess the relationship between serum IgE levels and the weight of insulin-positive or glucagon-positive cells in the sBBM Gimap5^+^/^+^ (Gimap5-DR) and Gimap5^−^/^−^ (Gimap5-DP) rats (no onset rats are included in the following analyses). To evaluate the predictive stability of serum IgE levels, we constructed four models for the weight of insulin-positive and glucagon-positive pancreas, incorporating different covariates to assess their impact. Subject ID was included as a random intercept to account for repeated measures. The outcome variable was the weight of insulin-positive or glucagon-positive pancreas (mg), with serum IgE levels as the predictor. The models included the following covariates:

Model 1: serum IgE levels (ng/ml) only. Model 2: serum IgE levels plus age. Model 3: serum IgE levels, age and genotype as a categorical variable with two levels (sBBM Gimap5^+^/^+^ [reference] and Gimap5^−^/^−^). Model 4: serum IgE levels, age, genotype, and glucose levels at the point of death. Model comparisons were conducted using the *performance* package in R, focusing on metrics such as AIC (with weights), AICc (with weights), BIC (with weights), Conditional R^2^, Marginal R^2^, Intraclass Correlation Coefficient (ICC), Root Mean Squared Error (RMSE), and Sigma. AIC and BIC values were used to assess model fit, with lower values indicating better fit, and model weights reflecting the relative likelihood of each model being the best. Conditional R^2^, which accounts for both fixed and random effects, and Marginal R^2^, which includes only fixed effects, were calculated to assess the variance explained by the models. Analyses and plots were conducted and generated using R and GraphPad.

## Results

**Fig. 2:**
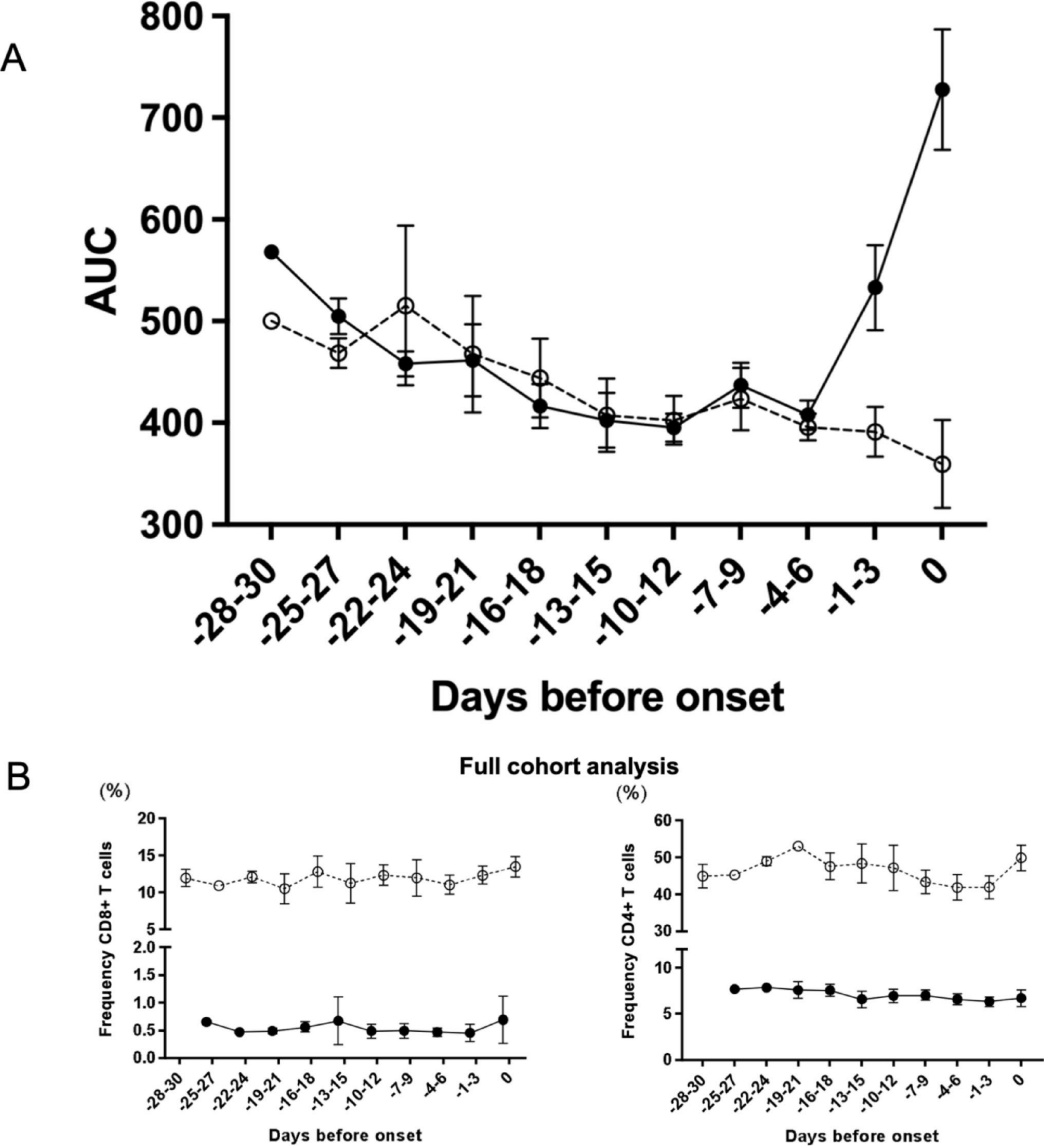
Longitudinal Assessment of Metabolic and Immunological Changes in BBM Gimap5-DP Rats Prior to Diabetes Onset. **A** OGTT was performed in BBM Gimap5^−^/^−^ rats (n = 29; filled circles) and control rats (n = 16, open circles, representing Gimap5-DR; Gimap5^+^/^−^ (n = 12) and Gimap5^+^/^+^ (n = 4) rats, as these two control genotypes did not differ significantly, they were analyzed as a single group). Two OGTT protocols were used: I) A 0–60 min OGTT was conducted in 18 BBM Gimap5^−^/^−^ (Gimap5-DP) rats and 12 controls (representing Gimap5-DR; 3 Gimap5^+^/^+^ and 9 Gimap5^+^/^−^ rats), II) a 0–90 min OGTT was conducted in 11 Gimap5^−^/^−^ (Gimap5-DP) rats and 4 control (Gimap5-DR; one Gimap5^+^/^+^ and 3 Gimap5^+^/^−^) rats. At 1 to 3 days prior to onset, the Area Under the Curve (AUC) from the 0–60 min OGTT showed a significant difference between BBM Gimap5^−^/^−^ (Gimap5-DP) and control (Gimap5-DR) rats (p = 0.036). When extended to 0–90 min, the AUC revealed a stronger significant difference (*p* = 0.000029). Data from OGTTs performed at 1 or 3 days before onset were combined for analysis. Data are presented as mean ± SEM. **B** Longitudinal flow cytometric analysis of CD8+ and CD4+ T cell frequencies in BBM Gimap5^−^/^−^ (Gimap5-DP) rats (filled circles, n = 10) and control (Gimap5-DR) rats (open circles, n = 11; 6 Gimap5^+^/^+^ and 5 Gimap5^−^/^+^). Data are presented as mean ± SEM. NB: Please note that time points of sample collection differ slightly between Gimap5^−^/^−^ (Gimap5-DP) and controls (Gimap5-DR) rats due to the availability of samples. Measurements are aligned to the day of diabetes onset in the DP group with, when possible, age-matched Gimap5-DR controls

**Fig. 3:**
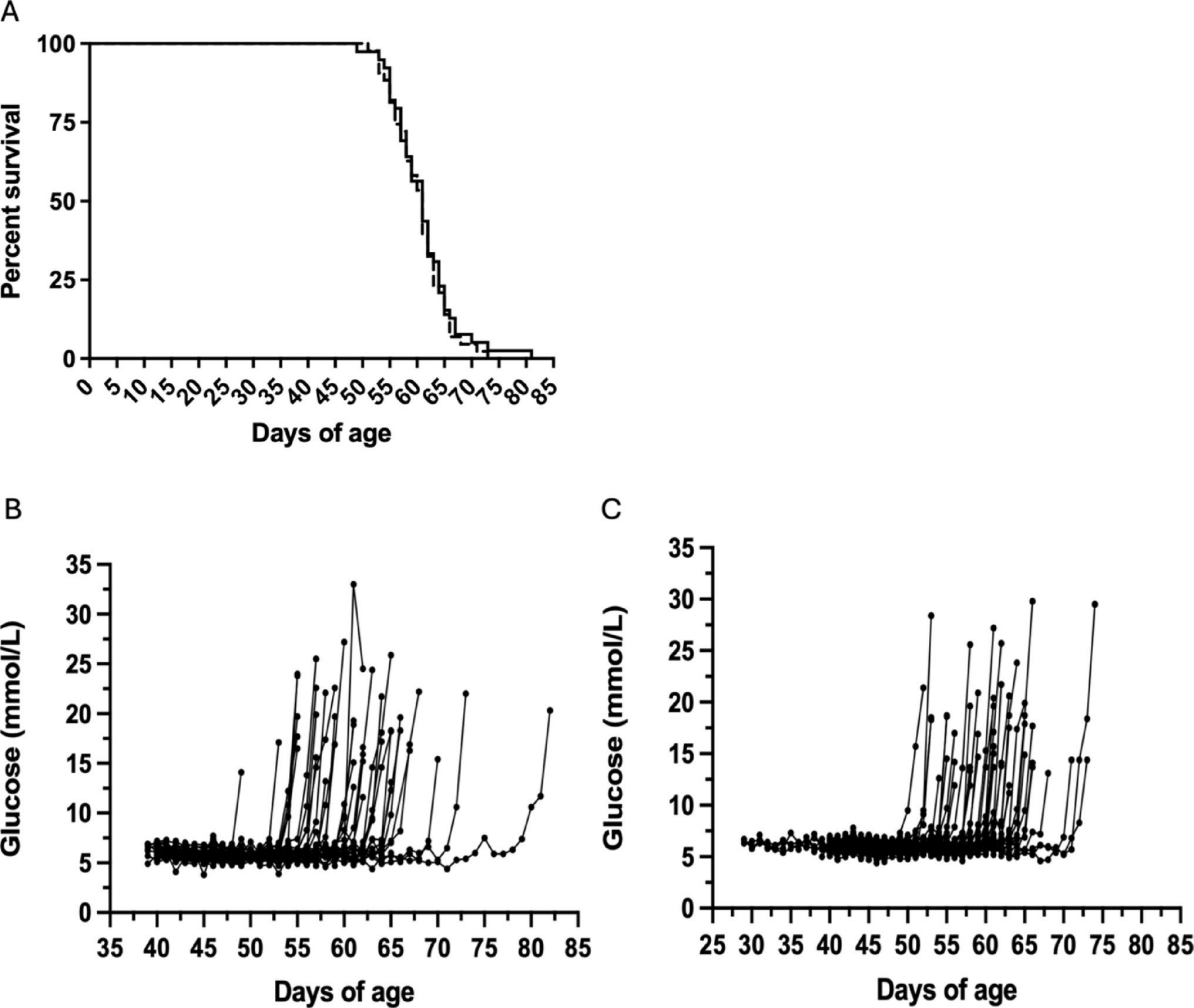
Impact of DP DNA Introgression Length on Diabetes Incidence and Blood Glucose Levels in Gimap5-DP Rats. **A** Cumulative diabetes incidence in Gimap5^−^/^−^ (Gimap5-DP) rats carrying either a full-length (1.44 Mbp) DP DNA introgression (BBM) or a shortened (1.02–1.26 Mbp) introgression (sBBM), showing no significant difference in onset. **B–C** Individual daily morning glucose measurements and glucose levels at diagnosis in BBM (**B**) and sBBM (**C**) Gimap5^−^/^−^ (Gimap5-DP) rats. Mean glucose at diagnosis: 17.3 ± 4.3 mmol/L (BBM) vs. 15.9 ± 3.2 mmol/L (sBBM); *p* = 0.105. Data presented as mean ± SD

The congenic BBM rats, derived from cross-intercross breeding (Fig. 1B), revealed a recombination event, which was fixed (Fig. 1A) and led to a reduction in DP contribution from approximately 1.44 (BBM) to 1.02–1.26 (sBBM) Mbp (Fig. 1A). Repeated OGTT detected impaired glucose tolerance three to one days prior to diabetes onset (Fig. 2A). The lymphopenia was reflected in the stable, reduced numbers of both CD4+ and CD8+ T cells, unaffected by diabetes development in the BBM Gimap5-DP rats (Fig. 2B). Using the R73 antibody as a pan-T cell marker, BBM Gimap5^−^/^−^ rats showed markedly fewer R73+ T cells (~ 12%) compared to wildtype and heterozygous littermates (~ 63%), indicating a significant reduction in T cell populations associated with the Gimap5 mutation. The cumulative incidence of diabetes (Fig. 3A) showed no difference between the two congenic lines. The median age at onset and range remained stable over time (Supplemental Table 1 ). Blood glucose levels at the time of clinical onset were comparable between the two lines (Fig. 3B-C). Growth (body weight in g or growth rate (g/day)) in the BBM did not differ from sBBM (data not shown). The sBBM rats were used to test the relationship between IgE levels and the pancreatic islet inflammatory process followed over time.

### Increased serum IgE in the sBBM Gimap5^−^/^−^ (Gimap5-DP) rats

**Fig. 4:**
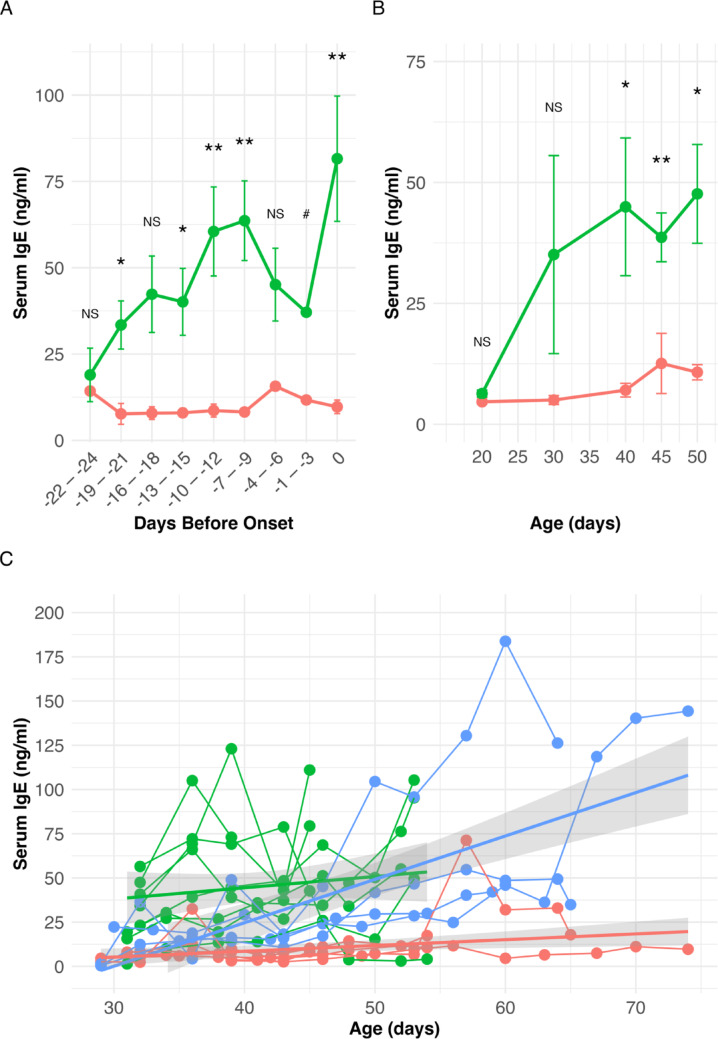
Longitudinal and age-related changes in serum IgE levels in sBBM Gimap5-DP and Gimap5-DR rats. **A** Longitudinal follow-up of serum IgE levels in sBBM Gimap5^−^/^−^ (Gimap5-DP, n = 9; green symbols) rats and Gimap5^+^/^+^ (Gimap5-DR, n = 7; red symbols) rats from 38 days of age until diabetes onset in the Gimap5^−^/^−^ (Gimap5-DP) group. Each rat was sampled at five timepoints. Statistically significant differences were observed at several time windows relative to diabetes onset: –21 to –19 days (*p* = 0.018), –15 to –13 days (*p* = 0.0158), –12 to –10 days (*p* = 0.00667), –9 to –7 days (*p* = 0.00191), and at day 0 (onset; *p* = 0.00410). A trend toward significance was seen from –18 to –16 days (*p* = 0.0509). Other time windows were not statistically significant. Data are presented as mean ± SEM. NS, not significant; *p < 0.05; **p < 0.01; # indicates time points with too few individuals for statistical testing. Statistical comparisons were performed using unpaired t-tests. **B** Age-stratified analysis of IgE levels (ng/mL) in sBBM rats sampled at various time points prior to diabetes onset. Data are shown for Gimap5^−^/^−^ (Gimap5-DP) rats (n = 5–9 per time point; green symbols) and Gimap5^+^/^+^ (Gimap5-DR) rats (n = 5–9 per time point; red symbols). Significant differences were observed at day 40 (*p* = 0.0373), day 45 (*p* 0.00756), and day 50 (*p* = 0.0109). Earlier time points were not statistically significant. Data are presented as mean ± SEM, and statistical comparisons were made using unpaired t-tests. NS, not significant; * *p* < 0.05; ** *p* < 0.01. **C** Comparison of IgE levels by disease stage and genotype: I) sBBM Gimap5^+^/^+^ (Gimap5-DR) rats (n = 6; red symbols); II) sBBM Gimap5^−^/^−^ (Gimap5-DP) rats prior to onset (n = 8; green symbols); III) sBBM Gimap5^−^/^−^ (Gimap5-DP) rats at the time of clinical onset (n = 4; blue symbols). Serum IgE was quantified using standardized ELISA assays. Results highlight dynamic, genotype-associated changes in IgE levels linked to progression toward diabetes.

In the longitudinal study, rats were bled every other day starting at 38 days of age until diabetes onset. Levels (µg/mL) of total serum IgE, IgG, IgA, and IgM were compared between sBBM Gimap5^+^/^+^ (Gimap5-DR, n = 7) and Gimap5^−^/^−^ (Gimap5-DP, n = 9) rats. As shown in Fig. 4A, serum IgE levels increased in Gimap5^−^/^−^ (Gimap5-DP) rats compared to controls, beginning approximately 20 days before diabetes onset. In contrast, no significant changes were observed in serum IgA, IgG, or IgM; these data are not shown.

By cross-sectional sampling, IgE was further analyzed in sBBM Gimap5^−^/^−^ (Gimap5-DP) rats killed between 20 and 50 days of age (n = 1–9 rats at each age) and at diabetes onset (Fig. 4B). IgE levels increased with age in both sBBM Gimap5^+^/^+^ (Gimap5-DR) and Gimap5^−^/^−^ (Gimap5-DP) (β = 0.7813, SE = 0.2968, t = 2.632, *p* = 0.010) but were significantly higher in the sBBM Gimap5^−^/^−^ (Gimap5-DP) rats compared to the Gimap5^+^/^+^ (Gimap5-DR) rats (β = 25.9810, SE = 6.3395, t = 4.098, *p* < 0.001). Additionally, the rate at which IgE increased with age tended to differ between sBBM Gimap5-DR and Gimap5-DP, approaching significance (p = 0.096). Eotaxin levels were associated with age (β = −9.066, SE = 4.072, t = −2.226, *p* = 0.029) but did not differ between sBBM Gimap5^−^/^−^ (Gimap5-DP) and Gimap5^+^/^+^ (Gimap5-DR) rats (β = −29.208, SE = 214.448, t = −0.136, *p* = 0.89) (Supplemental Fig. 1).

### Serum immunoglobulin isoform analysis shows elevated serum IgE levels in the sBBM Gimap5^−^/^−^ (Gimap5-DP) rats 

sBBM rats were monitored every other day for serum IgE levels. The rats were killed when serum IgE was elevated at 45 to 54 days of age, provided that their morning blood glucose remained normal (Fig. 4C).

sBBM Gimap5^−^/^−^ (Gimap5-DP) rats (n = 8; green symbols) were killed alongside age-matched Gimap5^+^/^+^ (Gimap5-DR, n = 6; red symbols) controls. An additional four rats were followed until the day of clinical diagnosis of diabetes as a disease control (blue symbols). Overall, sBBM Gimap5^−^/^−^ (Gimap5-DP) rats exhibited significantly higher IgE levels than matched Gimap5^+^/^+^ (Gimap5-DR) controls (*p* < 0.0001). Moreover, the rate at which IgE increased with age differed significantly between groups, with sBBM Gimap5^−^/^−^ (Gimap5-DP) rats showing a markedly steeper age-related rise compared to Gimap5^+^/^+^ (Gimap5-DR) controls (*p* = 0.0007) (Fig. 4C).

### Insulitis

Insulitis was evaluated and scored by two independent readers on coded slides stained with hematoxylin and eosin, revealing low-grade insulitis in the rats with elevated IgE but normal blood glucose levels. As expected, high-grade insulitis was observed in the newly diagnosed sBBM Gimap5^−^/^−^ (Gimap5-DP) rats (Supplemental Fig. 2).

### Infiltration analysis

**Fig. 5:**
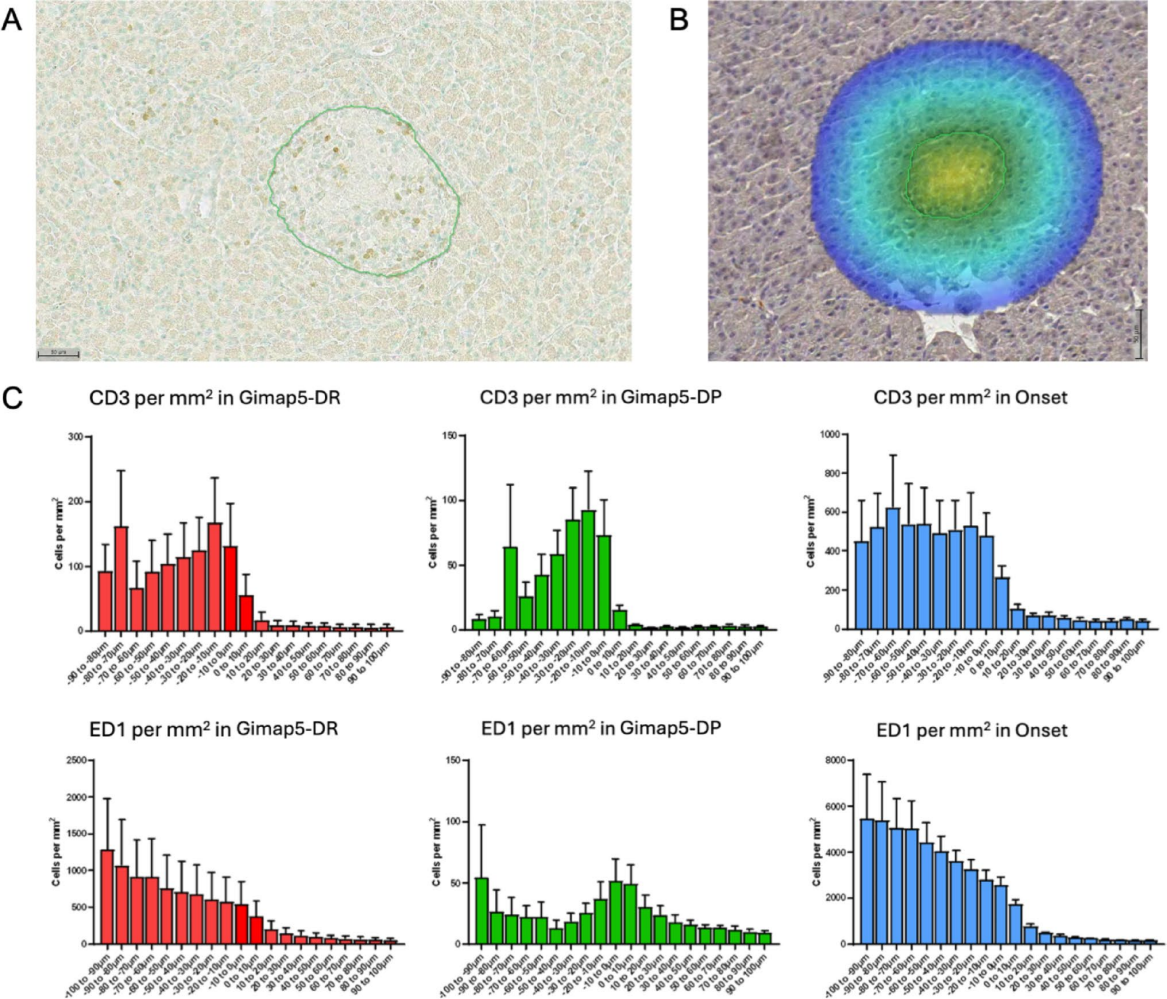
Spatial infiltration of CD3^+^ and ED1^+^ immune cells in pancreatic islets. **A** Representative image of CD3+ T cell infiltration in a sBBM Gimap5-DP rat. The perimeter of the analyzed islet is marked in green, defined using a convolutional neural network (CNN)-based segmentation algorithm. **B** Schematic illustrating the spatial analysis of infiltrating immune cells. The green line indicates the islet boundary. Analysis was performed in 10 μm intervals extending up to 100 μm into the islets interior (negative values) and 100 μm into the surrounding exocrine tissue (positive values), reltive to the islet perimeter (0 μm). **C** Quantification of infiltrating CD3+ T cells (top row) and ED1+ macrophages (bottom row) shown as cell density (cells/mm^2^) in 10 μm segments. Data are presented for sBBM Gimap5^+^/^+^ (Gimap5-DR) rats (n = 5; red bars), Gimap5^−^/^−^ (Gimap5-DP) prior to diabetes onset (n = 8; green bars), and Gimap5^−^/^−^ (Gimap5-DP) rats at the time of clinical onset of diabetes (n = 4; blue bars). Data are presented as mean ± SD

The infiltration of CD3+ and ED1+ cells was quantified by calculating the number of positive cells per mm^2^ within 10 μm distance bands spanning 100 μm both inside and outside the islet perimeter (Fig. 5A-B). To ensure special accuracy, islets were identified using a deep-learning classifier trained on CD3- and ED1-stained sections.

In sBBM Gimap5^+^/^+^ (Gimap5-DR) rats, CD3+ T cells accumulated more prominently toward the islet interior compared to ED1+ cells, which were more abundant in the surrounding exocrine tissue (Fig. 5C). In contrast, the Gimap5^−^/^−^ (Gimap5-DP) rats–characterized by lymphopenia (Fig. 2B)–displayed a distinct spike in CD3+ T cell density at the islet periphery, with little infiltration into the islet core. ED1+ cells in these rats were predominantly found outside the islets. In the newly onset diabetes rats, both CD3+ and ED1+ cells showed marked infiltration into the islets and a sharp decline in density in the exocrine tissue (Fig. 5C).

To assess spatial coordination, we analyzed correlation matrices of densities across the distance bands (Supplemental Fig. 3). In sBBM Gimap5^+^/^+^ (Gimap5-DR) rats, both immune cell types exhibited orderly spatial correlations—higher densities in one band were associated with similarly high densities in neighboring bands. In contrast, Gimap5^−^/^−^ (Gimap5-DP) rats showed a fragmented pattern, particularly for CD3+ cells, suggesting a more disorganized or diffuse infiltration pattern. No consistent associations were observed between IgE levels and infiltration across bands, although in Gimap5^+^/^+^ (Gimap5-DR) rats, ED1+ infiltration correlated with body weight.

### Image analysis of insulin and glucagon cells in relation to IgE levels

**Fig. 6:**
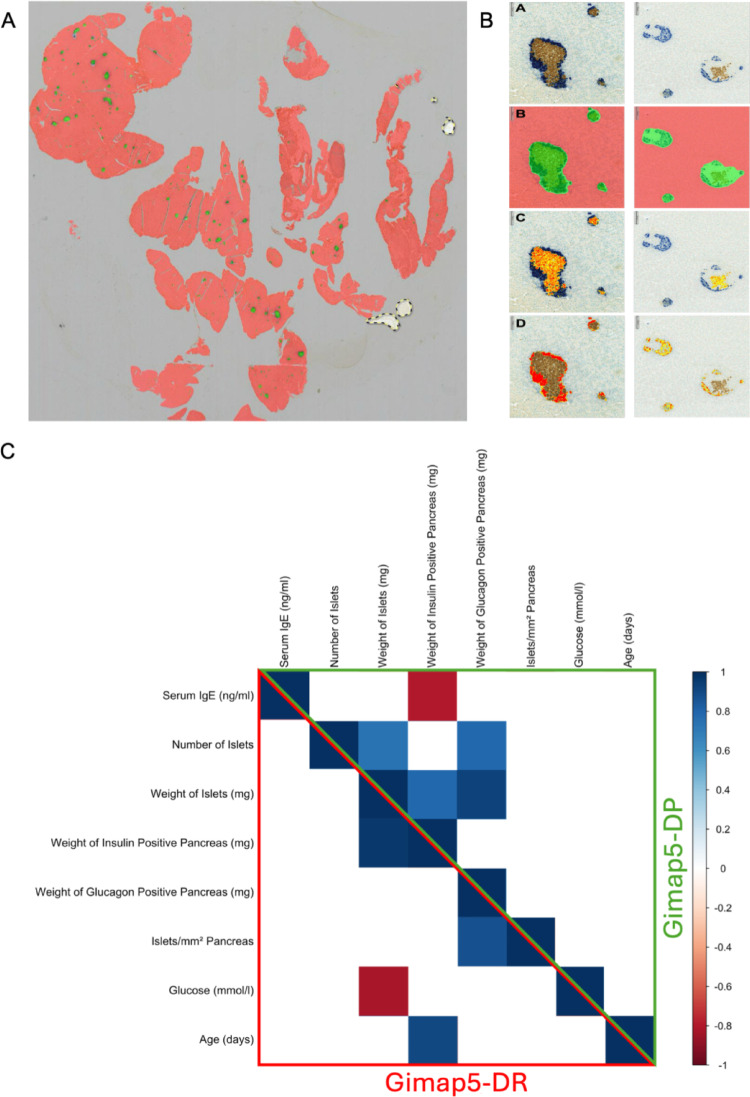
Relationship between IgE levels and immunohistochemical quantification of pancreatic islet cell composition. **A** Representative image of a pancreas section illustrating islet distribution used to quantify the total pancreatic area. Area Quantification Immunohistochemistry (AQ-IHC) was used to measure pixel-level intensity of brightfield stains for insulin and glucagon across whole-slide images. **B** Image panels demonstrate the immunohistochemical AI-assisted analysis for two representative islets. Row A: Original immunostaining for insulin (brown) and glucagon (blue) used for AI training; Row B: AI classification of exocrine tissue (red) and islets (green); Row C: Pixel-based quantification of insulin staining intensities (strong, moderate, weak); Row D: Pixel-based quantification of glucagon staining intensities. **C** Correlation matrix depicting relationships between IgE levels (ng/ml), number of islets, weight of islets (mg), the weight of insulin and glucagon-positive pancreas (mg), islets/mm^2^ pancreas (islet density), blood glucose levels (mmol/l), and age (days). sBBM Gimap5^+^/^+^ (Gimap5-DR) rats are shown in the red triangle, and sBBM Gimap5^−^/^−^ (Gimap5-DP) rats prior to diabetes onset in the green triangle. Only significant correlations (*p* < 0.05) are shown. Positive correlations appear in blue, negative in red

We next asked whether the IgE level was related to the immunohistochemical quantification of cells stained with antibodies to insulin and glucagon, respectively (Fig. 6). The islet quantification method allowed us to measure the quantity of insulin- and glucagon-positive cells (Fig. 6A-B). This enabled us to first relate IgE levels to the total amount in mg of both insulin (Supplemental Fig. 4A) and glucagon (Supplemental Fig. 4B) positive cells. Correlation analysis revealed a significant negative association between IgE levels and insulin-positive pancreas weight in Gimap5^−^/^−^ (Gimap5-DP) rats (r = –0.79, *p* = 0.019), but not in Gimap5^+^/^+^ (Gimap5-DR) rats (r = –0.16, *p* = 0.755) (Fig. 6C). Linear mixed-effects models confirmed this relationship, with IgE levels significantly predicting insulin-positive tissue area while controlling for age and genotype (Table 1 and in detail in Supplemental Table 2). No such association was found for glucagon-positive tissue.

**Table 1:** Fixed effects results from linear mixed models assessing the relationship between insulin or glucagon-positive pancreas weight and serum IgE levels

Pancreas	Model	Term	Estimate	95% CI
Insulin positive	1	Serum IgE	** − 0.0201**	[− 0.031, − 0.009]
	2	Serum IgE	** − 0.0151**	[− 0.022, − 0.008]
		Age	*0.0764*	[0.043, 0.109]
	3	Serum IgE	− 0.0172	[− 0.027, − 0.008]
		Age	*0.0782*	[0.045, 0.111]
		sBBM Gimap5^−^/^−^ (DP)	0.2374	[− 0.488, 0.963]
	4	Serum IgE	** − 0.0187**	[− 0.027, − 0.010]
		Age	*0.0785*	[0.050, 0.107]
		sBBM Gimap5^−^/^−^ (DP)	0.4426	[− 0.211, 1.096]
		Glucose	** − 0.3456**	[− 0.672, − 0.020]
Glucagon positive	1	Serum IgE	− 0.0002	[− 0.009, 0.009]
	2	Serum IgE	− 0.0007	[− 0.010, 0.009]
		Age	− 0.0078	[− 0.053, 0.037]
	3	Serum IgE	− 0.0026	[− 0.015, 0.010]
		Age	− 0.0061	[− 0.051, 0.039]
		sBBM Gimap5^−^/^−^ (DP)	0.2070	[− 0.786, 1.200]
	4	Serum IgE	− 0.0033	[− 0.016, 0.010]
		Age	− 0.0059	[− 0.050, 0.039]
		sBBM Gimap5^−^/^−^ (DP)	0.3023	[− 0.722, 1.327]
		Glucose	− 0.1578	[− 0.666, 0.350]

Different models were run, including various covariates, to assess the stability of the predictive capacity of serum IgE levels. Typographic coding: Bold indicates a significant negative association, while italics indicates a significant positive association with insulin or glucagon-positive pancreas weight (mg). Serum IgE is expressed in ng/ml, and glucose in mmol/l

### Islet autoantibodies

Comparable autoantibody binding levels between sBBM Gimap5^+^/^+^ (Gimap5-DR) and Gimap5^−^/^−^ (Gimap5-DP) rats showed no sign of autoantibodies for tGADA, GADA, IA-2A, and ZnT8A as measured by IgG-specific radiobinding assays (Supplemental Fig. 5). Please note that these assays may not detect insulin-specific IgE [16].

### Whole genome sequencing and genetic mapping

Mean coverage ranged from 31.4 to 48.5 across all chromosomes. The genetic map of BBM shows a total introgression of 1.44 Mbp of DP rat DNA (from SNP1 to SNP7, Table 2). The recombination in sBBM resulted in a reduction of DP rat DNA from 1.44 Mbp to a range of 1.02–1.26 Mbp, a decrease of 0.18–0.42 Mbp, as determined by comparing variant differences to the reference genome. The DP rat DNA contains 27–32 genes, ten of which differ in coding regions compared to the sBBM Gimap5^+^/^+^ (Gimap5-DR) rat (Supplemental Table 3). Three of these genes contain high-impact frame-shift variants: *Sspo*, *Gimap4*, and *Gimap5*.

**Table 2:** Candidate genes in the sBBM Gimap5-DR and Gimap5-DP rats

geneID	Name	From (bp)	To (bp)	sBBM Gimap5^+^/^+^ (DR)	sBBM Gimap5^−^/^−^ (DP)
ENSRNOG00000005310	Cul1	77211692	77280250	**2**	**2**
ENSRNOG00000006048	Ezh2	77284404	77347011	**2**	**2**
ENSRNOG00000061624	Y_RNA	77385900	77385977	**2**	**2**
ENSRNOG00000059013	lnc_RNA: AABR07060519.1	77413672	77446624	**2**	**2**
ENSRNOG00000056608	Y_RNA	77459794	77459895	**2**	***?***
ENSRNOG00000060430	Y_RNA	77463050	77463161	**2**	***?***
ENSRNOG00000006228	Pdia4	77470636	77489535	**2 (1)**	***? (1)***
ENSRNOG00000036459	miRNA: AABR07060522.1	77479040	77479116	**2**	***?***
ENSRNOG00000006925	Zfp786	77495663	77510202	**2**	**1**
ENSRNOG00000006703	Zfp398	77519028	77545589	**2**	**1**
ENSRNOG00000026981	Zfp282	77554269	77580714	**2**	**1**
ENSRNOG00000006711	Zfp212	77592016	77603226	**2**	*1 (1)*
ENSRNOG00000026874	Zfp956	77623538	77635362	**2**	*1 (1)*
	***SNP1***^***a***^	77630116	77630116	**2**	*1*
ENSRNOG00000033639	Zfp777	77684488	77706994	**2**	*1*
ENSRNOG00000007064	Zfp746	77723183	77747070	**2**	*1*
ENSRNOG00000007405	Krba1	78024765	78046066	**2**	*1 (1)*
ENSRNOG00000007707	Zfp467	78067689	78075398	**2**	*1 (1)*
ENSRNOG00000025848	Sspo	78080310	78133654	**2**	*1 (9)*
ENSRNOG00000024795	AC123494.1	78140483	78160638	**2**	*1 (10)*
ENSRNOG00000008218	Atp6v0e2	78168117	78171265	**2**	*1*
	***SNP2***^***a***^	78170204	78170204	**2**	*1*
ENSRNOG00000008234	Lrrc61	78187117	78206818	**2**	*1*
ENSRNOG00000024705	Rarres2	78205812	78208767	**2**	*1*
ENSRNOG00000052606	pseudogene: AC099444.3	78227687	78227801	**2**	*1*
ENSRNOG00000008239	Repin1	78233332	78236781	**2**	*1*
ENSRNOG00000008362	Zfp775	78240385	78258545	**2**	*1*
ENSRNOG00000024617	AC099444.1	78263866	78265056	**2**	*1*
ENSRNOG00000020169	Gimap8	78283026	78294845	**2**	*1*
ENSRNOG00000024569	Gimap9	78310379	78313206	**2**	*1*
ENSRNOG00000008369	Gimap4	78320190	78327144	**2**	*1 (1)*
ENSRNOG00000033338	Gimap6	78336423	78342863	**2**	*1*
ENSRNOG00000038740	Gimap7	78354335	78358503	**2**	*1*
ENSRNOG00000042229	Gimap1	78371121	78375223	**2**	*1 (1)*
	SNP3^***a***^	78377812	78377812	**2**	*1*
ENSRNOG00000008416	Gimap5	78378144	78385577	**2**	*1 (1)*
	SNP4^***a***^	78383990	78383990	**2**	*1*
	SNP5^***a***^	78386592	78386592	**2**	*1*
ENSRNOG00000008465	Tmem176b	78450724	78458179	**2**	*1*
ENSRNOG00000023708	Tmem176a	78458625	78462423	**2**	*1 (1)*
ENSRNOG00000008575	Aoc1	78496043	78515584	**2**	*1*
ENSRNOG00000023612	Doxl1	78563625	78567241	**2**	***?***
ENSRNOG00000053177	snoRNA	78571460	78571582	**2**	***?***
	SNP6^***a***^	78576514	78576514	**2**	**2**
ENSRNOG00000032432	Doxl2	78618737	78622523	**2**	**2**
ENSRNOG00000008714	Svs1	78639672	78644570	**2**	**2**
ENSRNOG00000054040	lncRNA: AC120721.1	78667427	78684552	**2**	**2**
ENSRNOG00000008816	Gpnmb	78694447	78715683	**2**	**2**
ENSRNOG00000009035	Malsu1	78735279	78744102	**2**	**2**
ENSRNOG00000009052	Igf2bp3	78746547	78879294	**2**	**2**
ENSRNOG00000009156	Tra2a	78905380	78924181	**2**	**2**
ENSRNOG00000034168	Gemin7l1	78954599	78954997	**2**	**2**
ENSRNOG00000009322	Ccdc126	78981987	79003053	**2**	**2**
ENSRNOG00000043400	LOC100910678	79015316	79015660	**2 (1)**	**2**
ENSRNOG00000009447	Fam221a	79021872	79043844	**2**	**2**
ENSRNOG00000009583	RGD1563352	79052157	79052633	**2**	**2**
ENSRNOG00000057554	Stk31	79055280	79153831	**2**	**2**
	SNP7^***a***^	79071296	79071296	**2**	**2**
ENSRNOG00000054527	lnc_RNA: AABR07060539.1	79184109	79185910	**2**	**2**
ENSRNOG00000043158	RGD1564712	79324582	79349893	**2**	**2**

The number in parenthesis is the number of variants found in that particular gene. Typographic coding: bold is DR, italic is DP, and bold italics is an area with few variants, uncertain if DR or DP

^a^ SNP1-7 refer to the genotyping PCR assays used to identify offspring in the heterozygous breeding to generate Gimap5^−^/^−^ (Gimap5-DP, 25%), heterozygous Gimap5^+^/^−^ (Gimap5-DR/DP, 50%) and Gimap5^+^/^+^ (Gimap5-DR, 25%) rats

The question mark (?) denote inconclusive sequence information

## Discussion

Our marker-assisted breeding to introgress DR genetic factors linked to *Gimap5* onto BBM Gimap5-DP rats demonstrates that the distal reduction of DP BB rat DNA from approximately 1.4 to 1.02 Mbp did not affect the diabetes and lymphopenia phenotypes. The first major conclusion of this study is that WGS revealed that none of the introgressed DR genes appeared to contribute to the diabetes and lymphopenia phenotypes. This observation supports previous findings that a diabetes and lymphopenia gene is more likely to be located if the introgression occurs proximal to the *Gimap5* locus [15].

The second major conclusion from this study is that while baseline serum IgE levels differed between groups, the most notable feature was the markedly steeper, age-related increase in IgE observed in sBBM Gimap5-DP rats. This accelerated trajectory may reflect underlying immune dysregulation and could potentially serve as an early indicator of islet-directed immune activity. Notably, elevated IgE levels preceded both a detectable rise in blood glucose and the onset of diabetes. During this prediabetic phase, CD3+ T cells—but not ED1+ macrophages—were found to increasingly infiltrate the islets, coinciding with a reduction in insulin-positive beta-cell mass. These findings suggest a temporal association between rising IgE levels, CD3+ infiltration, and beta-cell loss in this congenic rat model, although causality remains to be established. While these observations support the potential utility of IgE as a biomarker of early islet autoimmunity, it is important to acknowledge that evidence from clinical studies is mixed. Elevated IgE levels have been reported in T1D patients and may reflect disease heterogeneity or contribute to pathogenesis [21, 22, 40]. However, some studies—particularly in insulin-treated patients—highlight limitations in interpreting insulin-specific IgE due to low-affinity antibodies or localized binding [41]. As our study does not involve insulin administration or antigen-specific IgE measurements, these concerns are not directly applicable, but they underscore the need for context-specific validation. Ultimately, our findings in the sBBM rat model support further investigation into total and antigen-specific IgE responses as early markers or modulators of autoimmune diabetes. Additionally, the Area Quantification Immunohistochemistry provided data demonstrating that the total insulin content, measured in milligrams per milligram of pancreas, was associated with body weight loss, supporting previous studies [42, 43]. The potential to use the present approach to the human pancreas has limitations. Rat islets exhibit a core-mantle structure, where the core is predominantly composed of beta cells, while the surrounding mantle contains alpha and delta cells. In contrast, human islets are more heterogeneous, with beta, alpha, and delta cells interspersed throughout the islet. While this distinction highlights the relevance of the observed infiltration into the core of rat islets, where beta cells are primarily located, other factors must be considered when investigating the human pancreas.

Since 1980, the original DP rat has undergone marker-assisted cross-intercross breeding with inbred DR rats [44] to introgress DR DNA. This process aimed to dissect the mechanisms by which homozygous BB DR Major Histocompatibility Complex (MHC) RT.1B^u/u^ interacts with the *Gimap5* deletion to induce early lymphopenia and diabetes at 50–70 days of age [5, 14, 45–47]. The observed diabetes in BB rats has been related to lymphopenia [48] and is found to segregate with diabetes as a single locus [45]. The lymphopenia gene was cloned by position [14, 49], demonstrating a frame-shift mutation in the *Gimap5* gene, resulting in a null allele of the anti-apoptotic Gimap5 protein [15, 50].

The genes that differ between DP and DR are listed in Table 2 and detailed in Supplemental Table 3 as potential “diabetes-lymphopenia genes”. Our previous analysis of recombination events that reduced the contribution of DP, which was replaced by DR DNA, suggested that a “diabetes gene” would be located proximal to the Gimap5 mutation [15]. First, the introgression of a 33-Mb region of the F344 genome, proximal to the mutated Gimap5 gene, renders the rat diabetes resistant despite having lymphopenia [51]. Second, further breeding to yield additional recombinations identified T cell receptor *beta 8E*, *12*, and *13* as candidate genes in one region, and *Znf467* and *Atp6v0e2* as candidate genes in a second region closer to the *Gimap5* locus. Thus, spontaneous diabetes may be controlled by at least two genetic loci, 7 Mb apart on rat chromosome 4 [15]. The present analysis suggests that *Atp6v0e2* is an unlikely candidate, as our WGS analysis did not detect a difference between DP and DR. Conversely, *Znf467* showed at least two missense mutations (Table 2, Supplemental Table 3) and remains a potential contributing factor. Repin1 has previously been identified as a genetic factor contributing to high-fat diet protection against diabetes in the OK/BB rats [52], while the present genetic analysis did not reveal a difference between DP and DR *Repin1* (Table 2).

Our analysis suggests that there are three genes in the remaining DP region with high impact differences between sBBM Gimap5-DR and Gimap5-DP. In addition to *Gimap5*, it is important to note that *Gimap4* has a frameshift mutation (Supplemental Table 3) that alters the C-terminal end of the expressed protein, as previously reported [53]. Interestingly, Gimap4 has been identified as a hypomorphic variant, an AT(+) variant in approximately 30% of rats, including wild rats [54]. Therefore, *Gimap4* appears to represent a candidate genetic factor that may contribute to lymphopenia and diabetes.

An important feature of the sBBM Gimap5-DP rats is their inability to produce any of the islet antigen autoantibodies that are strongly associated with human type 1 diabetes [55, 56]. The now available WGS should prove useful for further analyzing genetic factors in the MHC region, which may be important for understanding the origin of MHC-associated beta-cell killing. A strength of the present study is the HALO™-based quantitative analysis of infiltrating CD3+ and ED1+ cells, as well as insulin- and glucagon-positive cells. The HALO™ image analysis software was previously used to quantify insulin and glucagon cells, as well as hyaluronic acid as an early marker of insulitis [8, 13]. However, what is novel to this investigation is the attempt to discern whether ED1+ (macrophages and dendritic) cells infiltrate the pancreas and the islets before the CD3+ cells. RelB+ dendritic cells were detected before ED1+ cells prior to the clinical onset [5]. Although we did not perform longitudinal tracking, the spatial distribution of immune cells across disease stages and strains provides insight into potential infiltration patterns. In diabetes-resistant sBBM Gimap5-DR rats, CD3+ T cells were observed within the islet interior, where beta cells are centrally located in rats, while ED1+ macrophages remained largely peripheral. The presence of CD3+ cells in this location may reflect physiological immune surveillance rather than active autoimmunity. However, their proximity to beta cells suggests that if autoreactivity emerges—as in Gimap5-DP rats—this natural localization could facilitate early beta-cell-directed immune responses. In the lymphopenic Gimap5-DP rats, CD3+ cells accumulated mainly at the islet border, and ED1+ cells were largely excluded, consistent with impaired infiltration due to reduced T cell numbers. Despite this limitation, the observed pattern may still reflect early antigen recognition at the islet interface.

The apparent early presence of CD3+ cells in the islet interior prior to clinical onset of diabetes (Supplemental Fig. 3) may shift our understanding of insulitis progression. Rather than macrophages or dendritic cells leading the infiltration, our data suggest that CD3+ T cells may be the first to engage the islet. The subsequent infiltration of ED1+ macrophages may represent a secondary response, potentially involved in clearing beta cell debris following a CD8+ T cell-mediated attack.

Importantly, flow cytometric analysis using the R73 antibody—recognizing approximately 97% of peripheral rat T cells—revealed a marked reduction of R73+ T cells in lymphopenic Gimap5-DP rats (~ 12%) compared to Gimap5-DR rats (~ 63%). This decrease reflects a substantial reduction in circulating T cells consistent with lymphopenia, although absolute total lymphocyte counts were not directly measured in this study. A detailed analysis of leukocyte numbers have been reported previously [57]. Therefore, while the Gimap5-DP rats show a clear T cell deficiency indicative of lymphopenia, further studies are needed to confirm total lymphocyte counts below the clinical threshold (< 1,500 cells/μL). It is important to note that despite this T cell deficiency, we observe a significant CD3+ infiltration pattern of the pancreatic islets prior to clinical onset and a prominent infiltration at the time of clinical onset (Fig. 5).

A limitation of the quantitative immunohistochemistry performed is that the multiplex immunocytochemistry technique did not always work, and adjacent sections were analyzed using single-plex staining. The technical challenges of achieving multiplex staining prevented simultaneous staining for CD3+ , CD8+ , and CD4+ cells, which would have provided valuable insights into the character of T-cell infiltration in relation to IgE levels. Another weakness is that the infiltrating cells were analyzed around the islets rather than in conjunction with veins, arteries, and ducts.

The unexpected finding that *Gimap5*, along with *Gimap4*, may regulate diabetes development in the BB rat is likely to translate into human type 1 diabetes autoimmunity. Both genes have been implicated in lymphocyte development and immune regulation. GIMAP5 is essential for T cell survival and homeostasis; its deficiency leads to impaired maturation and survival of CD4+ and CD8+ T cells, as well as defective NK and NKT cell development, and is associated with immune dysfunction and liver pathology in both rodents and humans [58–61]. GIMAP4 has been implicated in T cell biology and was found to be aberrantly activated in T-cell acute lymphoblastic leukemia, supporting a functional role in lymphocyte regulation [62]. *GIMAP5* may be involved in the pathogenesis of systemic lupus erythematosus [63]. We found a SNP located in the polyadenylation signal of *GIMAP5* associated with levels of IA-2 autoantibodies [64]. *GIMAP4* was associated with allergic sensitization, while *GIMAP5* was linked to asthma [65]. Gene–gene interactions were noted between *GIMAP4* and *IL2RA*, a known human type 1 diabetes risk gene [66], as well as between *GIMAP5* and *INS*, a major type 1 diabetes risk gene [67] strongly associated with insulin autoantibodies as the first appearing islet autoantibody [68, 69]. It was suggested that GIMAP4 and GIMAP5 apoptosis regulator proteins may modify immune-mediated diseases [65]. These apoptosis regulator proteins are novel and represent potential targets for disease-modifying therapeutics in humans.

New directions regarding how cells infiltrate the pancreas and the islets may focus on identifying which type of CD3+ cells are the first to infiltrate the islets. The remaining segment of DP DNA is about 1.02–1.26 Mbp. While the present study identified candidate genes within this region through WGS analysis, it cannot be excluded that future cross-intercross breeding may result in additional recombination events. Further investigation of gene expression within these candidate regions will help clarify their potential involvement. We conclude that there are a limited number of genetic factors, including but not limited to *Gimap4* and *Gimap5,* that are related to the increase in IgE levels, insulitis, infiltration of CD3+ and ED1+ cells, and beta cell loss prior to diabetes onset in congenic sBBM Gimap5-DP rats.

## Supplementary Information

Below is the link to the electronic supplementary material.

Supplementary Material 1

Supplementary Material 2

## Data Availability

The raw sequencing reads and associated metadata for BBDP.BBDR-(*Zfp786-Aoc1*)/Ulund, RRID: RGD_597830140 (sBBM) have been deposited in the European Nucleotide Archive (ENA) under accession number PRJEB85825 ([https://www.ebi.ac.uk/ena]) in compliance with FAIR principles, ensuring accessibility with rich metadata and interoperability through standard formats. The code used for analyses is available upon request.
