# Supplementary material for: Quantitative temporal analysis of pancreatic islet T lymphocyte and macrophage infiltration heralded by serum IgE in congenic BioBreeding (BB) Gimap5−/− rats at risk for insulitis and acute onset diabetes
Source: Inflamm Res. 2025 Oct 3;74(1):134. doi: 10.1007/s00011-025-02101-9 (PMC12494634; doi:10.1007/s00011-025-02101-9)
Supplement: Supplementary file 1 — Supplementary Material 1 [file 11_2025_2101_MOESM1_ESM.docx]

*Supplemental Material*

Quantitative temporal analysis of pancreatic islet T lymphocyte and macrophage infiltration heralded by serum IgE in congenic BioBreeding (BB) Gimap5-DP rats at risk for insulitis and acute onset diabetes.

Josefine Jönsson^*^, Linda Faxius^*^, Jeanette Tångrot, Krysten Vance, Stephanie Jerman, Doug Bowman, Marika Bogdani, Peter Ericsson, Rasmus Bennet, Anita Ramelius, and Åke Lernmark.

*Shared first authorship

Corresponding author:

Josefine Jönsson, PhD

Lund University CRC

Department of Clinical Sciences

Jan Waldenströms gata 35,

SE-214 28 Malmö, Sweden

Tel: +46 40 39 19 01

[josefine.jonsson@med.lu.se](mailto:josefine.jonsson@med.lu.se)

*Supplemental Material Legends:*

**Supplemental Table 1.** Number of Gimap5^-^/^-^ (Gimap5-DP) rats born and followed until diabetes onset in the Lund University CRC SPF facility in Malmö, Sweden.

**Supplemental Table 2.** Detailed results of fixed effects from linear mixed models, including model comparison. (Excel file)

**Supplemental Table 3.** Detailed differences in candidate genes that distinguish DR from DP.

**Supplemental Fig. 1** Relationship between eotaxin and age in sBBM Gimap5-DP (green symbols) and DR.Gimap5-DR (red symbols) rats.

**Supplemental Fig. 2** Insulitis scores in pancreas sections stained with hematoxylin and eosin (H&E) in relation to IgE levels.

**Supplemental Fig. 3** Correlation of immune cell infiltration patterns in sBBM Gimap5-DR and Gimap5-DP rats.

**Supplemental Fig. 4** Relationship between IgE levels and insulin- or glucagon-positive islet cell mass.

**Supplemental Fig. 5** Longitudinal analysis of islet autoantibodies in BBM Gimap5-DP and DR.Gimap5-DR rats.

**Supplemental Table 1. Number of** Gimap5^-^/^-^ (Gimap5-DP) **rats born and followed until diabetes onset in the Lund University CRC SPF facility in Malmö, Sweden.**

| **Year** | **BBM (n)** | **Diabetes (%)** | **Median age**  **(days)** | **Range**  **(days)** |
| --- | --- | --- | --- | --- |
| **2008-2010** | 131 | 100 | 63 | 48-78 |
| **2011-2013** | 9* | 78 | 60 | 51-70 |
| **2014-2016** | 21 | 100 | 61 | 54-73 |
| **2017-2019** | 49 | 100 | 60 | 49-71 |
| **2020-2022** | 5 | 100 | 59 | 52-58 |
| **2023** | 6 | 100 | 62 | 57-64 |
| **Total** | **221** | **96** | **61** | **48-78** |
|  | **sBBM (n)** |  |  |  |
| **2015-2017** | 33 | 100 | 61 | 51-73 |
| **2018-2020** | 5 | 100 | 62 | 53-66 |
| **2021-2023** | 13 | 100 | 59 | 53-72 |
| **Total** | **51** | **100** | **61** | **51-73** |

* An involuntary *Streptococcus aureus* infection episode reduced diabetes frequency.

**Supplemental Table 2. Detailed results of fixed effects from linear mixed models, including model comparison.** Models were fitted to assess the relationship between weight of insulin or glucagon-positive pancreas (mg) and serum IgE (ng/ml), with individual variation accounted for as a random effect (1 | ID). Covariates were included or excluded as needed. Model comparison was conducted using the performance package in R, evaluating fit using criteria such as AIC, BIC, and conditional/marginal R². **Color coding:** Red indicates a significant negative association, while green indicates a significant positive association with insulin or glucagon-positive pancreas weight (mg).

Excel file

**Supplemental Table 3. Detailed differences in candidate genes that distinguish Gimap5^+^/^+^ (Gimap5-DR) from Gimap5^-^/^-^ (Gimap5-DP).**

| Location (bp) | Gene id /  Gene | SNP id | Codon change | Aa* | Type** |
| --- | --- | --- | --- | --- | --- |
| 77 472 708 | ENSRNOG00000006228 /  Pdia4 | rs13448732 | cGa/cAa | R/Q | missense |
| 77 602 420 | ENSRNOG00000006711 /  Zfp212 | rs197708173 | Tca/Gca | S/A | missense |
| 77 633 571 | ENSRNOG00000026874 /  Zfp956 | rs105708158 | aAg/aCg | K/T | missense |
| 78 041 619 | ENSRNOG00000007405 /  Krba1 | rs106932336 | Tcc/Ccc | S/P | missense |
| 78 044 011 | ENSRNOG00000007405 /  Krba1 | rs104948091 | cAg/cGg | Q/R | missense |
| 78 070 043 | ENSRNOG00000007707 /  Zfp467 | rs65569384 | Tca/Aca | S/T | missense |
| 78 070 745 | ENSRNOG00000007707 /  Zfp467 | rs64494067 | Ccc/Tcc | P/S | missense |
| 78 083 676 | ENSRNOG00000025848 /  Sspo | rs197040130 | Aca/Gca | T/A | missense |
| 78 084 516 | ENSRNOG00000025848 /  Sspo |  | Ccg/Tcg | P/S | missense |
| 78 084 520 | ENSRNOG00000025848 /  Sspo |  | acc/  acTTTTATGTGTc | T/  TFMCX | frameshift |
| 78 089 681 | ENSRNOG00000025848 /  Sspo | rs198757490 | aTa/aCa | I/T | missense |
| 78 094 101 | ENSRNOG00000025848 /  Sspo | rs107066471 | Tgg/Cgg | W/R | missense, splice_region_variant |
| 78 094 212 | ENSRNOG00000025848 /  Sspo | rs106976086 | Gtc/Atc | V/I | missense |
| 78 097 074 | ENSRNOG00000025848 /  Sspo | rs197771394 | Gtg/Atg | V/M | missense |
| 78 099 423 | ENSRNOG00000025848 /  Sspo | rs107361784 | tCg/tTg | S/L | missense |
| 78 100 315 | ENSRNOG00000025848 /  Sspo | rs106099788 | aAt/aGt | N/S | missense |
| 78 146 486 | ENSRNOG00000024795 /  AC123494.1 | rs197949628 | cCg/cTg | P/L | missense |
| 78 158 617 | ENSRNOG00000024795 /  AC123494.1 | rs106681355 | Gtg/Atg | V/M | missense |
| 78 158 645 | ENSRNOG00000024795 /  AC123494.1 | rs104946449 | gAa/gGa | E/G | missense |
| 78 159 514 | ENSRNOG00000024795 /  AC123494.1 | rs106078095 | Agt/Ggt | S/G | missense |
| 78 159 595 | ENSRNOG00000024795 /  AC123494.1 | rs106362890 | Agt/Ggt | S/G | missense |
| 78 159 640 | ENSRNOG00000024795 /  AC123494.1 | rs197480311 | Cgt/Tgt | R/C | missense |
| 78 159 674 | ENSRNOG00000024795 /  AC123494.1 | rs198812103 | gCc/gTc | A/V | missense |
| 78 159 739 | ENSRNOG00000024795 /  AC123494.1 | rs198181341 | Agt/Ggt | S/G | missense |
| 78 159 786 | ENSRNOG00000024795 /  AC123494.1 | rs106480428 | atT/atG | I/M | missense |
| 78 159 787 | ENSRNOG00000024795 /  AC123494.1 | rs106132409 | Atg/Ctg | M/L | missense |
| 78 326 657 | ENSRNOG00000008369 /  Gimap4 |  | ATa/a | I/X | frameshift |
| 78 375 047 | ENSRNOG00000008416 /  Gimap5 | rs198740515 |  |  | upstream_gene_variant |
| 78 383 990 | ENSRNOG00000008416 /  Gimap5 |  | Ccc/cc | P/X | frameshift |
| 78 459 760 | ENSRNOG00000008465 /  Tmem176b |  |  |  | upstream_gene_variant |

*Aa: is amino acid change. **Type: type of mutation.


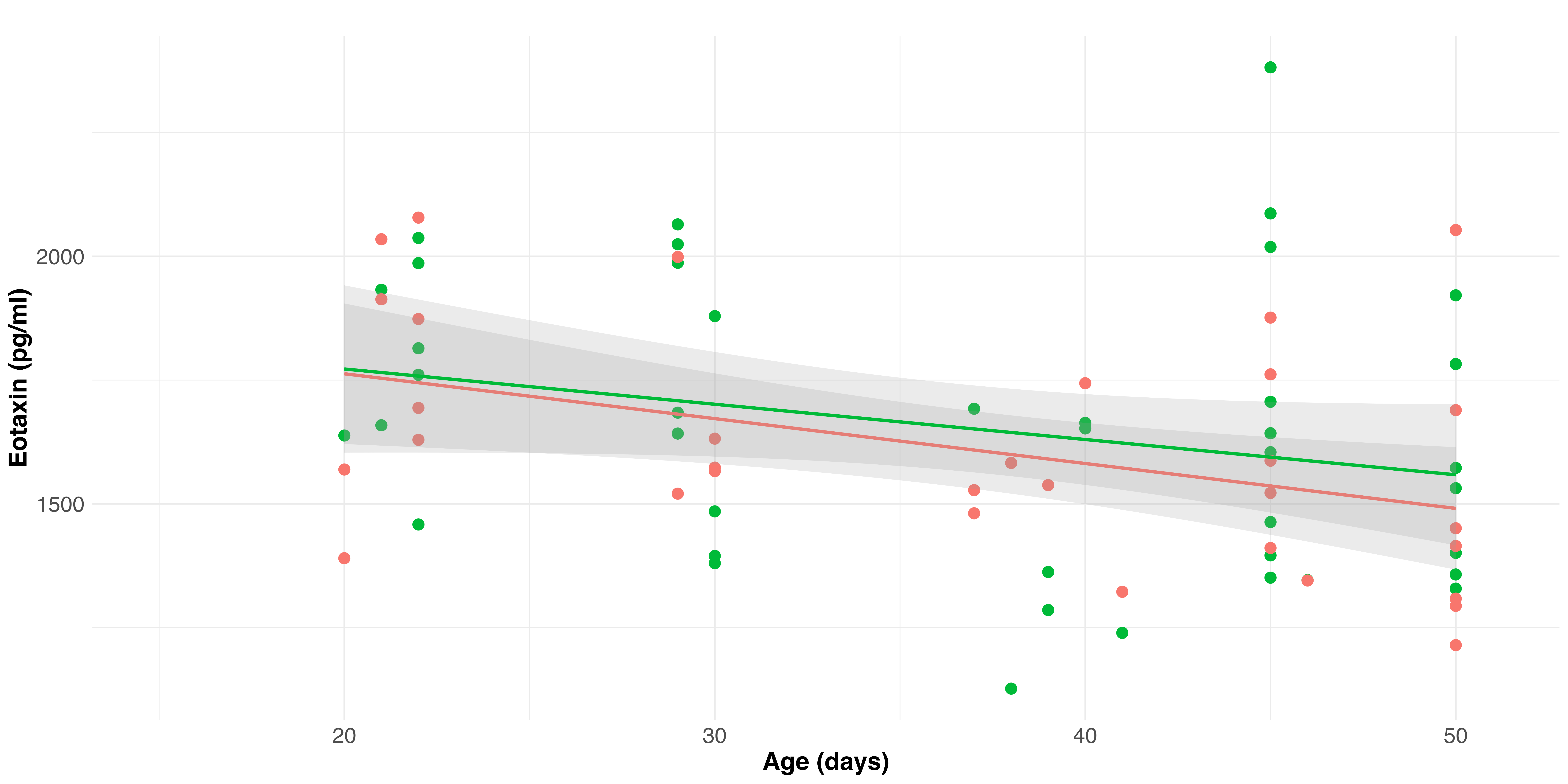


**Supplemental Fig. 1 Relationship between eotaxin and age in sBBM Gimap5^-^/^-^ (Gimap5-DP, green symbols) and Gimap5^+^/^+^ (Gimap5-DR, red symbols) rats.** Eotaxin levels (pg/ml) at different ages prior to diabetes onset in the Gimap5^-^/^-^ animals. N=5-9 rats per age group in both Gimap5^-^/^-^ (Gimap5-DP) and Gimap5^+^/^+^ (Gimap5-DR) rats.


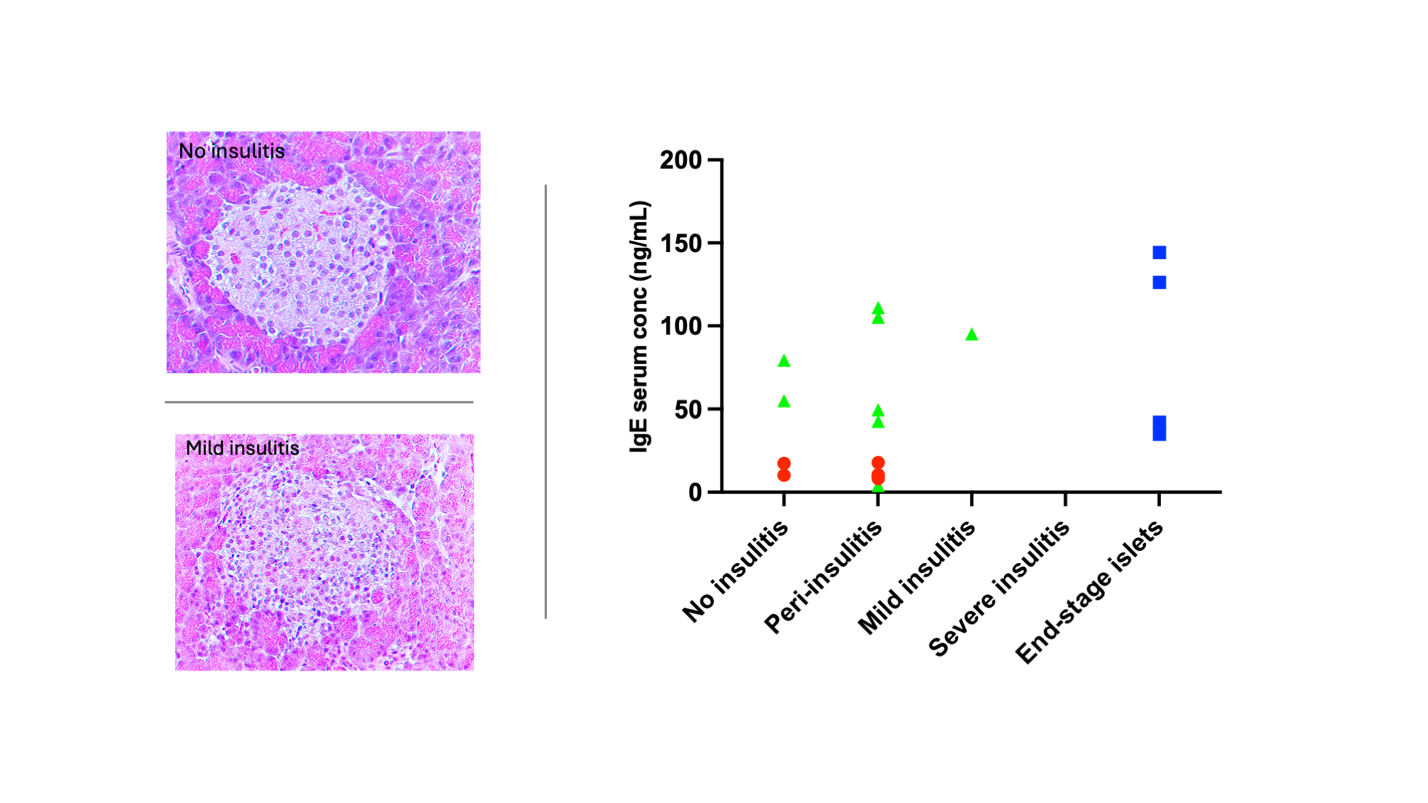


**Supplemental Fig. 2 Insulitis scores in pancreas sections stained with hematoxylin and eosin (H&E) in relation to IgE levels.** Insulitis was evaluated in three groups of rats I) sBBM Gimap5^+^/^+^ (Gimap5-DR) rats (n=5; red circles), serving as non-diabetic controls. II) Gimap5^-^/^-^ (Gimap5-DP) rats analyzed prior to clinical diabetes onset but exhibiting elevated IgE levels (n=8, green triangles) and III) Gimap5^-^/^-^ (Gimap5-DP) rats at the time of clinical onset (n=4; blue squares). IgE levels were measured in parallel to assess their relationship with insulitis severity. Pancreatic sections were stained with H&E, and insulitis was scored histologically.


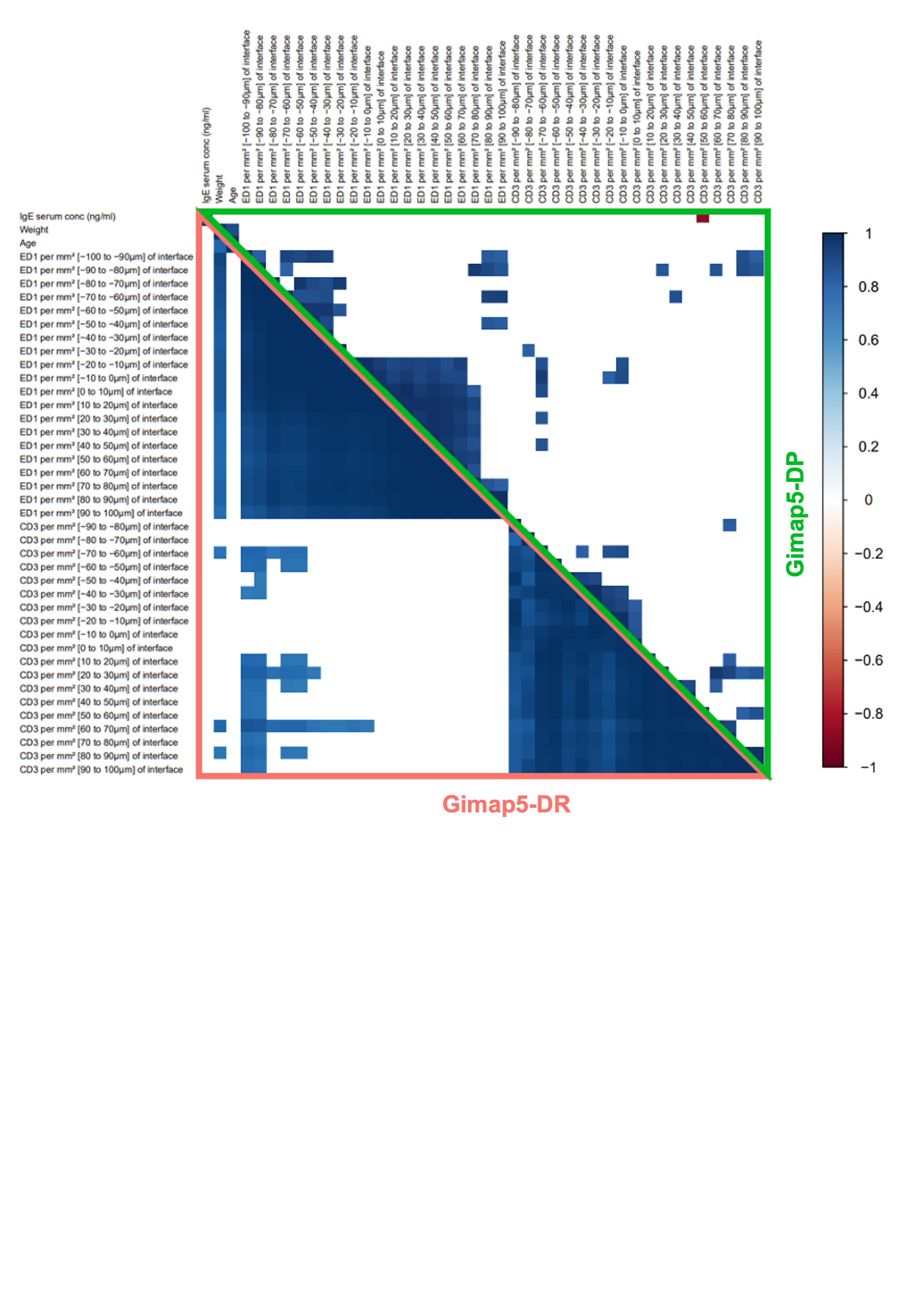


**Supplemental Fig. 3 Correlation of immune cell infiltration patterns in sBBM Gimap5^+^/^+^ (Gimap5-DR) and Gimap5^-^/^-^ (Gimap5-DP) rats.** Correlation matrix of cell densities for infiltrating CD3⁺ T cells and ED1⁺ macrophages across 10 µm segments spanning from the islet interior (negative values) to the exocrine tissue (positive values), relative to the islet boundary (0 µm). sBBM Gimap5^+^/^+^ (Gimap5-DR) rats are shown in the red triangle, and Gimap5^-^/^-^ (Gimap5-DP) rats prior to diabetes onset in the green triangle. Only statistically significant correlations (p < 0.05) are displayed, with positive correlations in blue and negative correlations in red.

Notably, the sBBM Gimap5-DR rats exhibited an orderly correlation pattern across both cell types, while the Gimap5-DP rats showed a fragmented and disorganized matrix—suggestive of disrupted immune infiltration dynamics, potentially associated with elevated IgE levels (see Fig. 5A).


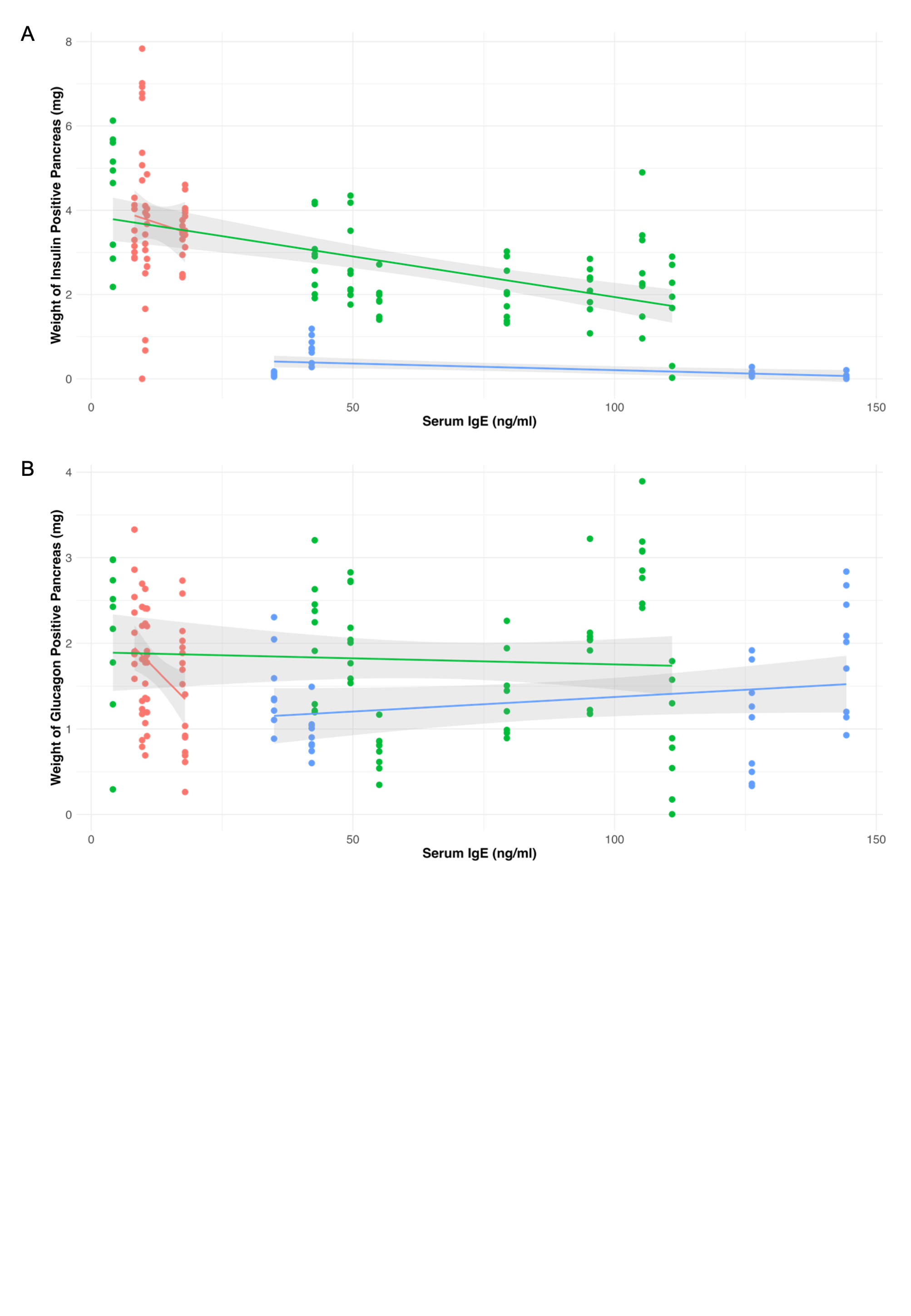


**Supplemental Fig. 4 Relationship between IgE levels and insulin- or glucagon-positive islet cell mass.**

**A** Serum IgE levels (ng/mL) plotted against the weight of insulin-positive cells per mg of pancreas in: sBBM Gimap5^+^/^+^ (Gimap5-DR) rats (n = 6; red symbols); sBBM Gimap5^-^/^-^ (Gimap5-DP) rats prior to diabetes onset (n = 8; green symbols), sBBM Gimap5^-^/^-^ (Gimap5-DP) rats at clinical onset (n = 4; blue symbols). All serial sections from each animal were included in the quantification. **B** Same as panel A but showing the relationship between IgE levels and the weight of glucagon-positive cells per mg of pancreas in the same groups of rats.

**Supplemental Fig. 5 Longitudinal analysis of islet autoantibodies in BBM Gimap5-DP and Gimap5-DR rats.** Islet autoantibodies were measured over time in two groups: I) BBM Gimap5^-^/^-^ (Gimap5-DP) rats (n = 9; filled circles) and Gimap5^+^/^+^ (Gimap5-DR) rats (n = 7; open circles). The following autoantibodies were analyzed: **A** full-length GAD autoantibodies (GADA); **B** truncated GADA (lacking the N-terminal 95 amino acids); **C** IA-2 autoantibodies (IA-2A), and; **D** ZnT8 autoantibodies (ZnT8A). Data are presented as mean ± SEM.
